# Fabrication and Characterization of Diaphragm Headphones Based on Graphene Nanocomposites

**DOI:** 10.3390/ma17040933

**Published:** 2024-02-17

**Authors:** Shun-Fa Hwang, Hsien-Kuang Liu, Wei-Chong Liao, Yi Kai Cheng

**Affiliations:** 1 Department of Mechanical Engineering, National Yunlin University of Science and Technology, 123 University Road, Sec. 3, Douliu, Yunlin 64002, Taiwan; hwangsf@yuntech.edu.tw; 2 Department of Mechanical and Computer Aided Engineering, Feng Chia University, 100 Wenhwa Road, Taichung 40724, Taiwan; 3 Department of Civil Engineering, Feng Chia University, 100 Wenhwa Road, Taichung 40724, Taiwan; wcliao@fcu.edu.tw; 4 Graduate School of Natural Science and Technology, Okayama University, 3-1-1 Tsushimanaka, Kita-ku, Okayama 700-8530, Japan; sz6505@gmail.com

**Keywords:** graphene oxide paper, nanocomposite, sound pressure level, compliance, line-indented pattern, curve-indented pattern

## Abstract

The goal of this paper is to fabricate innovative diaphragm headphones using graphene oxide paper (GOP) and GOP/epoxy nanocomposites (GOPC). Initially, graphene oxide suspension is fabricated, and the vacuum filtration method is adopted to make GOP. Then, vacuum bag molding is used to fabricate GOPC from GOP. Hot pressing and associated molds are adopted to fabricate line-indented (GOPC-L) or curve-indented patterns (GOPC-C) on the GOPC. The performances of one kind of GOP and three kinds of GOPC diaphragm headphones are analyzed based on their sound pressure level (SPL) curves achieved by the Soundcheck measurement system. There are four important processing parameters that will influence the performance of the diaphragm, including material type GOP versus GOPC, indented pattern type, sonication time on suspension, and graphene weight fraction in suspension. Compliances of various diaphragms are measured by the Klippel LPM laser measurement system. The results indicate that effects of sonication time and graphene weight fraction on SPL of GOP and GOPC headphones are in reverse, and this is associated with their difference on compliance (modulus), mass, damping ratio, and microstructure uniformity. Either GOPC-L or GOPC-C seems to improve the microstructure of the GOPC, and leads to better SPL performance. The correlation between the previous four factors and SPLs of four kinds of diaphragm headphones is proposed by using scanning electron microscope (SEM) to examine the microstructure of these diaphragms.

## 1. Introduction

Graphene, the one-atom-thick strong bonded carbon membrane, exhibits intriguing electronic, thermal, mechanical, and optical properties. By stacking a few layers of graphene nanosheets, the material becomes graphene papers (GPs). GPs can preserve most properties that monolayer graphene possesses. Initially, Dikin et al. [1] fabricated GO (graphene oxide) paper from GO water solution by a vacuum filtration method. The GO paper had a tensile strength of 120 MPa. Suk et al. [2] first fabricated monolayer graphene by a CVD method and then transferred this graphene film onto PET (polyethylene terephthalate) as graphene/PET nanocomposite film to be a loudspeaker. A sound wave was created to the surrounding air by the nanocomposite film due to thermoacoustic effect, and the sound pressure level (SPL) of this film was around 50 dB. Xu [3] found that the piezoelectric PVDF film could be sandwiched by two graphene layers. Once the voltage was applied on graphenes, the reverse piezoelectric effect could lead to the vibration of PVDF film and sound waves were created. A few layers of graphene were grown on the surface of the polar X-cut (110) of a piezoelectric La_3_Ga_5.5_Ta_0.5_O_14_ crystal using the CVD method [4], which indicates a good matching of crystal lattice parameters between piezoelectric and two-dimensional graphene crystals. This hybrid material has high potential to apply as an advanced acoustic headphone.

Kim et al. [5] used nitrogen-doped reduced graphene oxide arrays (N-rGOA) to make a thermoacoustic array loudspeaker. A new type of flexible headset (earphone) based on graphene using a laser direct writing method was introduced by Ren et al. [6]. Compared to the traditional magnetic headphones, this headset could capture subtle sounds in ultrasonic bandwidth, which could be applied in the field of human and animal communication.

Headphone performance is closely related to the vibration behavior of diaphragms. The vibration of graphene sheets via a 2D nanomechanic plate model was studied [7,8,9,10]. They concluded that the deflection in the vibration of graphene sheets was nonlinear and the amplitude was large. Mirakhory et al. [11] investigated the vibration behavior of pristine and defective triangular graphene sheets. They found that by increasing the resonance range of sensors based on 2D graphene sheets, the triangular structure was better than the square structure. The vibration and damping characteristics of graphene nanoplatelet (GNP)-modified epoxy composites were studied [12,13,14]. The results showed that the damped natural frequencies in GNPs/epoxy composites decreased with the addition of GNPs nanofillers due to the decrease in the toughness of nanocomposites. With the addition of 0.4 wt% GNPs, an increase of 26% in the damping ratio of p-GNPs/epoxy composite was observed.

Based on the literature review, graphene papers are excellent diaphragm candidates for acoustic devices due to their high stiffness and low mass. The aim of this paper is to fabricate graphene oxide paper (GOP) from well-dispersed graphene oxide (GO) nanosheet water suspension using the vacuum filtration method. In addition, graphene oxide paper/epoxy nanocomposites (GOPC) are fabricated by the vacuum bag molding method. Then, graphene oxide paper (GOP), and GOP/epoxy nanocomposites (GOPC) with a GO weight fraction of 26% are adopted as diaphragms in the electromagnetic headphones. In addition, lined-pattern (GOPC-L) and curved-pattern (GOPC-C) indentations are molded onto both surfaces of the diaphragm by hot-press molding. Therefore, there are four important parameters affecting the acoustic performance of the diaphragm, including the material type (GOP versus GOPC), pattern type on nanocomposites (line or curve), sonication time (T1~3), and graphene weight fraction in suspension (WF1~3) for GOP. Sound pressure level (SPL) curves of GOP and GOPC diaphragm headphones are analyzed by the Soundcheck measurement system. Compliances of the diaphragms are measured by the Klippel LPM laser measurement system.

## 2. Materials and Methods

Graphene oxide (GO) nanosheets were fabricated by the modified Hummers method. First, 12 g expanded graphite powders were added to 460-mL H_2_SO_4_ and the suspension was stirred in an ice bath. Later, 60 g of KMnO_4_ were slowly added to the suspension, heated to 35 °C, and stirred for 2 h. Then, 920 mL of deionized water was slowly added in the suspension and the temperature had to be kept under 50 °C. The suspension was further diluted in 2800 mL deionized water, and then 50 mL of 30% concentration H_2_O_2_ was added. The suspension stood still for 24 h. After that, the suspension was initially set in a centrifuge by a revolution of 6000 rpm for 1 h to separate the solute and solvent. The solute was further purified to be GO by the addition of the mixture of water and methanol. The centrifuge and purification processes were repeated for several cycles.

GO papers (GOP) were fabricated by the vacuum filtration method. GO suspension was poured into the upper container of a vacuum filtration apparatus. The lower bottle had an outlet that connects to a vacuum pump. There was a filtration assembly consisting of porous glass and cellulose filter paper between the upper and lower parts of the apparatus. When the vacuum pump was turned on, the solvent of the GO suspension was sucked out and GO nanosheets were combined to be GOP above the filtration assembly. After the drying process, a free-stand circular GOP with diameter of 35 mm can be achieved.

To fabricate GOP/epoxy nanocomposites (GOPC), a vacuum bag molding method was adopted. First, a GOP was soaked into epoxy resin at 60 °C for 30 min. Then, the soaked GOP covered by release fabrics was placed inside a vacuum bag, and a vacuum pump was connected to the outlet of the bag and started up. At the same time, the vacuum bag and epoxy resin-soaked GOP were placed inside an oven at 120 °C for 2 h to cure the epoxy. After the curing process, the GOPC diaphragm was achieved.

To fabricate patterned GOPCs, the initial steps for epoxy soaking and vacuum pumping for GOP were similar to those of GOPCs. Then, uncured GOP/epoxy was sandwiched between two patterned molds, either lined or curved patterns, and sprayed with the release agent. The molds were further set up between hot pressing platens with suitable pressure at 120 °C for 2 h. Thereafter, GOPC-L and GOPC-C diaphragms were achieved.

In order to clarify the effect of processing parameters on the acoustic performance of various GOP and GOPC diaphragms, Table 1 shows the terminology for various fabricated diaphragms. Sonication times on suspension for T1, T2, and T3 are 1, 5, and 10 min, respectively; GO weight fractions in suspension for WF1, WF2, and WF3, are 0.5, 1.0, and 1.5 wt%, respectively; pattern types on nanocomposites are lined (L) and curved (C) indentations, respectively.

**Figure 1:**
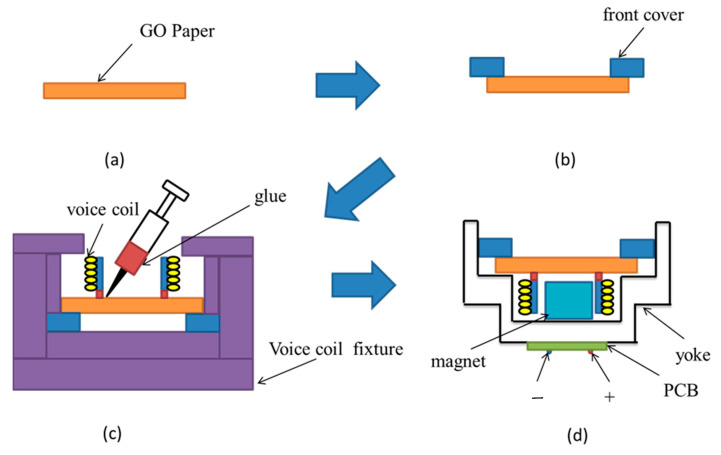
The fabrication procedure for a GOP headphone: (**a**) preparation of GOP, (**b**) adhesion of GOP on a plastic ring, (**c**) alignment and adhesion of GOP/voice coil, (**d**) assemblage of the GOP diaphragm with a headphone unit.

To manufacture an electromagnetic GOP diaphragm headphone, a plastic ring frame was initially adhered on the edge of the GOP to be a front cover, as shown in Figure 1a,b. Then, a dynamic voice coil was adhered on the opposite surface of the GOP, and centers of both voice coil and GOP must be aligned by a specific fixture (Figure 1c). Further, this diaphragm was assembled with a headphone unit consisting of a yoke, a magnet, and a PCB (Figure 1d). Positive and negative electrodes of the dynamic voice coil were connected to the corresponding electrodes of the PCB, and the headphone was ready for testing. The preparation procedure for the other three diaphragm headphones was similar.

**Figure 2:**
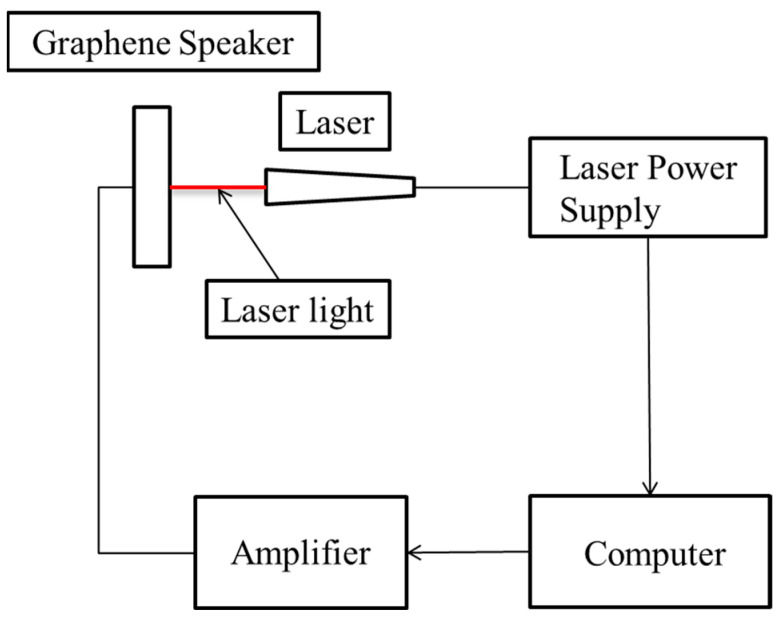
Setup of the Klippel LPM laser measurement system for compliance of the GOP diaphragm.

The SPL of the headphone was tested inside an anechoic chamber. A B&K 2716C amplifier was connected to the headphone and sound waves in the frequency range from 100 to 20 kHz were created. The sound waves were detected by a B&K 4191 microphone. The GOP and GOPC headphones were mounted on a baffle board based on an IEC 60268-5 regulation [15]. The distance between the headphones and the microphone was 2 cm. The input voltage of the speaker was 1.73 mV. The amplifier and microphone were connected to a computer with Soundcheck software (Version 15.03), and the SPL curve of the graphene diaphragm headphones was measured. To measure the compliance of the diaphragms, as shown in Figure 2, the set-up of the measurement system was similar to that for SPL, except the microphone was replaced by a Klippel LPM laser measurement system. Both GOP and GOPC diaphragms are first cut as suitable samples by diamond knife, and then coated with gold. Later they are put inside the chamber for scanning electron microscope (SEM) observation. SEM is used to carefully observe the microstructure of four types of diaphragms and correlated with their SPLs.

## 3. Results

### 3.1. Effect of Material Type

**Figure 3:**
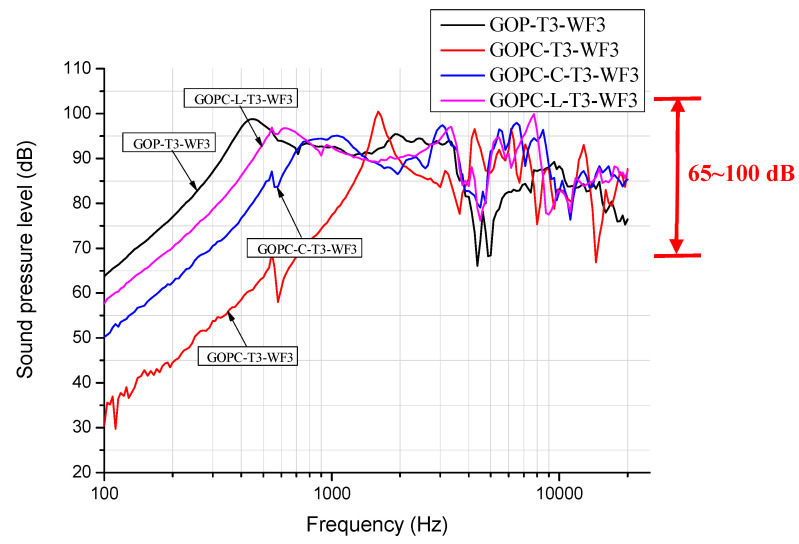
SPL curves for diaphragm headphones fabricated at highest sonication time of T3 and WF3 by GO papers (GOP), GOP/epoxy nanocomposite without a pattern (GOPC), and with lined (GOPC-L) and curved patterns (GOPC-C).

Figure 3 shows SPL (sound pressure level) curves for diaphragm headphones fabricated by GO papers (GOP) as well as GOP/epoxy nanocomposites without patterns (GOPC) and with the curve (GOPC-C) and line (GOPC-L)-indented patterns. All diaphragms were fabricated with GO weight fraction of 1.5 wt% (WF3) in suspension and with a sonication time of 10 min (T3). At frequencies lower than 3000 Hz, The GOP-T3-WF3 diaphragm has a smoother SPL curve than the other three nanocomposite diaphragms, GOPC-T3-WF3, GOPC-C-T3-WF3, and GOPC-L-T3-WF3. In addition, GOP possesses the largest-frequency bandwidth for SPL value above 90 dB, which is between 350 and 3.5 kHz, and the highest initial SPL value of 64 dB at a sweeping frequency of 100 Hz, which indicates that its sensitivity is good. This 90 dB threshold value of the GOP is equivalent to that required for the commercial diaphragm; in addition, the approximate flat curve between 2 k to 3.5 kHz could provide good sensitivity for human hearing. The smoother SPL curve of GOP is due to its higher mechanical compliance, C_ms_ of 7.25 m/N, as shown in Table 2. The highest SPL value for GOP is 98 dB at the first resonant frequency of 430 Hz, which is better than 50 dB in reference [2]. For the nanocomposite GOPC with the lower mechanical compliance of 0.3 m/N (higher stiffness), the first resonant frequency shifts to a higher frequency of 1800 Hz as compared with GOP, and its bandwidth is smallest.

Table 3 shows the maximum SPL values of four kinds of diaphragm headphones. The GOP headphone has the maximum SPL value of 99 dB and the other three kinds of nanocomposite diaphragm headphones possess maximum SPL values higher than 100 dB. All these results indicate that adopting GOP as diaphragm headphones is a viable route. In addition, as comparing the SPLs in Table 3 with the compliances in Table 2, the maximum SPL value seems to be inversely proportional to the compliance value. This could be attributed to the better specific modulus (E/ρ) of the GOP driver compared with that of the commercial beryllium copper (BeCu) thin-film driver [16,17,18]. The moduli E of the GOP and BeCu films were 3.41 GPa and 102 GPa, respectively, while the densities ρ of GOP and BeCu films were 0.15 and 8.25 g/cm^3^, respectively. Therefore, their specific moduli could be calculated as 22.73 (3.41/0.15) and 12.36 (102/8.25), respectively, which show that GOP has a significantly higher specific modulus than that of BeCu films. Actually, the specific modulus is proportional to sound speed ($\sqrt{E/\rho }$), and better specific modulus of GOP could result in good SPL performance.

**Figure 4:**
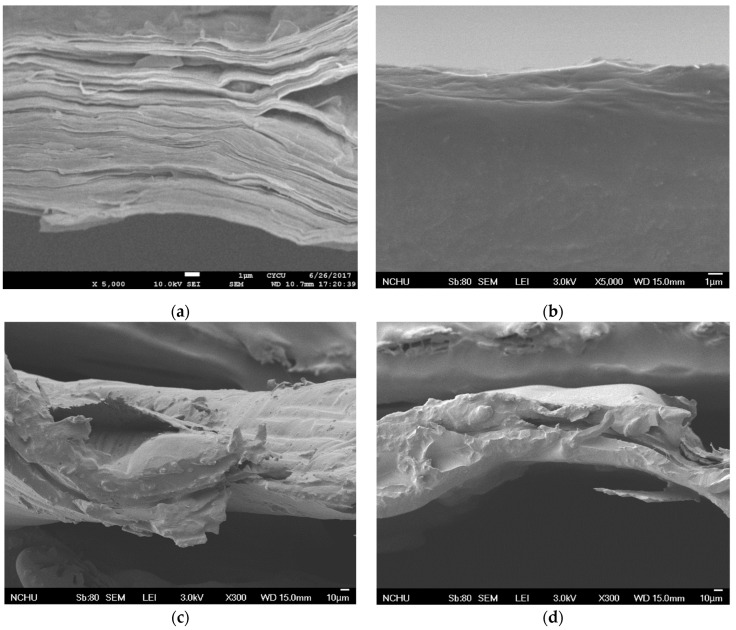
Micrographs of diaphragms (**a**) GOP-T3-WF3, (**b**) GOPC-T3-WF3, (**c**) GOPC-C-T3-WF3 and (**d**) GOPC-L-T3-W3.

Figure 4 depicts micrographs for the cross-sectional views of four kinds of diaphragms fabricated at the same processing parameters of T3 and WF3, and Figure 4a,b show those for GOP and GOPC, respectively. As shown in Figure 4a, thick and almost uniform stacked GO nanosheets were found and could be due to the highest GO weight fraction WF3. Only few interlaminar pores are found at the right hand side of the micrograph. On the other hand, more interlaminar holes exist for GOPC, as shown in Figure 4b, and this indicates that there is very little epoxy infiltration. Since the GOPC is fabricated by vacuum-bag molding, epoxy is very difficult to infiltrate in the through-thickness direction via tortuous pores inside the stacking 2D nanosheets of the GOP. More pores in the GOPC lead to its lower dB value at the initial sweeping frequency, as shown in Figure 3. Figure 4c,d show micrographs for the patterned GOPC-C and GOPC-L diaphragms, respectively. Two features are found in these two micrographs: first, both GOPCs depict a curve shape and the GOPC-C seems to possess the shape with a smaller radius of curvature at the left hand side of Figure 4c; second, both GOPCs show few intralaminar pores, indicating that epoxy infiltration is improved by the help of indentation process. Therefore, both features lead to medium compliance values and a more uniform microstructure. Their correlated SPLs are shown in Figure 3, and the corresponding discussion is proposed in Section 3.2.

### 3.2. Effect of Pattern on the Nanocomposite

**Figure 5:**
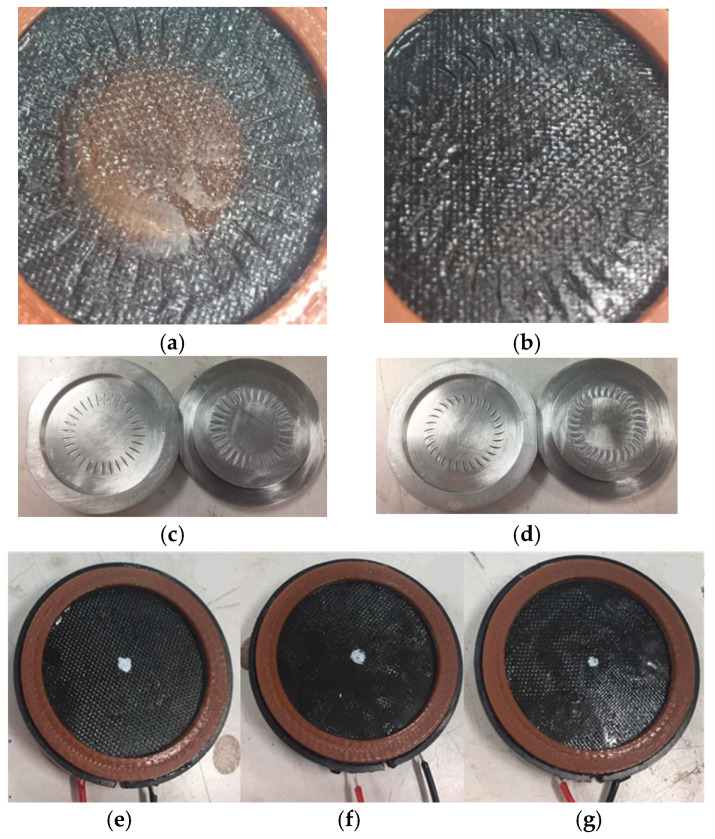
Photographs for fabricated nanocomposite diaphragm drivers and headphones: (**a**) driver GOPC-L, (**b**) driver GOPC-C, (**c**) line-patterned molds, (**d**) curve-patterned molds, and (**e**) headphone GOPC-T1-WF3, (**f**) headphone GOPC-T2-WF3, (**g**) headphone GOPC-T3-WF3.

Figure 5a,b depicts, respectively, the fabricated nanocomposite diaphragm drivers with lined (GOPC-L) and curved (GOPC-C) patterns. Both patterns are uniform short line or arc indentations in the radial direction fabricated, respectively, by the molds shown in Figure 5c,d. Figure 5e–g depict, respectively, the well-fabricated headphones, GOPC-T1-WF3, GOPC-T2-WF3, and GOPC-T3-WF3. Figure 3 also shows the effect of the two pattern types of nanocomposites on the SPL curves of diaphragm headphones. Due to higher compliances, both GOPC-L and GOPC-C headphones show higher SPL values at the initial 100 Hz compared with GOPC, and also indicate smoother SPL curves for frequencies higher than 10 kHz resulted from more uniform microstructure. With patterns on nanocomposite diaphragms, the first resonant frequencies of both GOPC-C and GOPC-L shift back to lower values due to the increase in mechanical compliance to medium values, as compared with that of GOPC. In addition, it is found that both patterned GOPCs could significantly improve the SPL performance of the GOPC diaphragm headphone in the lower-frequency range because the compliances of the former are higher than that of the latter. As shown in Table 2, the compliances C_ms_ of GOPC-L, GOPC-C, GOPC, and GOP were, respectively, 1.14, 0.88, 0.30, and 7.25 m/N. In the high-frequency range of larger than 3000 Hz, as shown in Figure 3, all four diaphragms depict unstable SPL curves; however, GOPC-L shows a relatively smoother SPL curve. Furthermore, the SPL performance of GOPC-L with higher compliance is better than that of GOPC-C in the low-frequency range, because the larger lateral vibration amplitude of the former contributes better to acoustic performance.

### 3.3. Effect of Sonication Time

**Figure 6:**
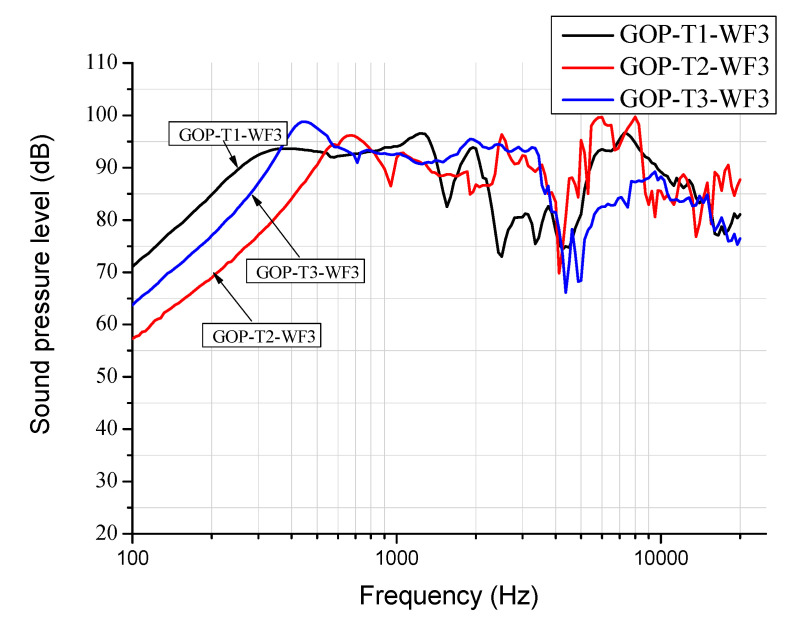
SPL curves for GO paper diaphragm headphones fabricated by various sonication times on suspension.

Figure 6 shows the SPL curves for GOP-WF3 diaphragm headphones fabricated by various sonication times T1~T3 (1~10 min) applied in suspension. For lower sonication time T1, the first resonant frequency of diaphragm GOP-T1 seemed to shift to a lower frequency of 300 Hz; in addition, in the frequency range from 300 to 1050 Hz, GOP-T1 also indicates a comparatively smoother curve. By the lower sonication time T1 applied in GO suspension, the pristine 2D GO nanosheets were broken and their size was larger compared with that by T3, and when these GO nanosheets combined as GOP they tended to have a loose and interlocked stacking. On the other hand, for GOP-T3 with higher T3, there is a tight packing of smaller GO nanosheets in the GOP with intrinsic small pores, indicated in Figure 4a. Upon frequency sweeping by SPL measurement in the high-frequency range around 10 kHz, those pores might lead to the oscillation of the SPL curve. However, this common oscillation phenomenon is called mode splitting, occurring even in the commercial diaphragm, which can be improved by an increased modulus of the diaphragm.

**Figure 7:**
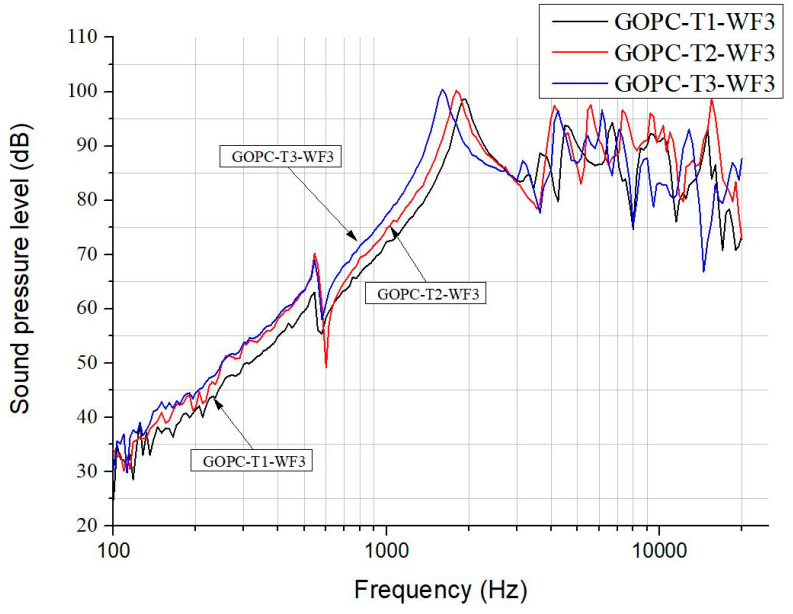
SPL curves for GOP/epoxy diaphragm headphones fabricated by various sonication times on suspension.

**Figure 8:**
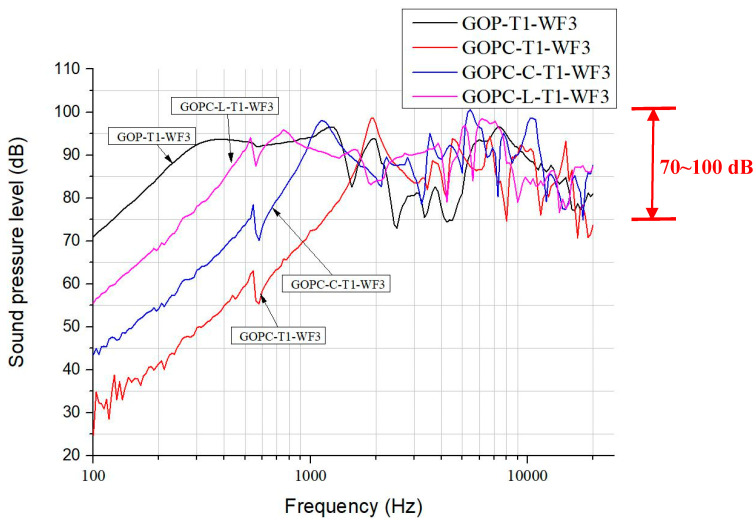
SPL curves for diaphragm headphones fabricated at lowest sonication time of T1 and WF3 by GO papers (GOP), GOP/epoxy nanocomposite without a pattern (GOPC), and with lined (GOPC-L) and curved patterns (GOPC-C).

Figure 7 depicts the SPL curves for GOPC-WF3 diaphragm headphones fabricated by various sonication times. The trend of the effect of sonication time on SPL for GOPC is in reverse as compared with that for GOP. GOPC-T3 has a wider bandwidth at lower frequencies and a smoother curve at higher frequencies than GOPC-T1. Fabricated by T3, GOP is stacked by smaller-sized 2D GO nanosheets, and pores inside GOP are less tortuous in the through-thickness direction. Upon fabricating from GOP to be GOPC, epoxy resin is better at infiltrating GOP. This results in better interlayer bonding of GOPC-T3 as well as better SPL performance. Figure 8 shows the four SPL curves for diaphragm headphones fabricated at the lowest sonication time of T1 and WF3 by GOP, GOPC, and patterned GOPC-L and GOPC-C. This figure can be compared with Figure 3 for the four headphones fabricated by T3. For diaphragms fabricated by T1, GOP-T1-WF3 had a higher initial SPL value of 71 dB at 100 Hz than that of GOP-T3-WF3. Either T1 or T3 may lead to different diaphragms having the highest SPL higher than 100 dB, for GOPC-C-T1-WF3 occurring at 5500 Hz, while for GOPC-T3-WF3 occurring at 1500 Hz. In the high-frequency range, more than 2 kHz, by T1 the oscillation extent of SPL curves of the diaphragms ranged between 70 to 100 dB, as shown in Figure 8, which was less significant than that by T3 in the range of 65 to 100 dB, as shown in Figure 3.

### 3.4. Effect of Graphene Weight Fraction

**Figure 9:**
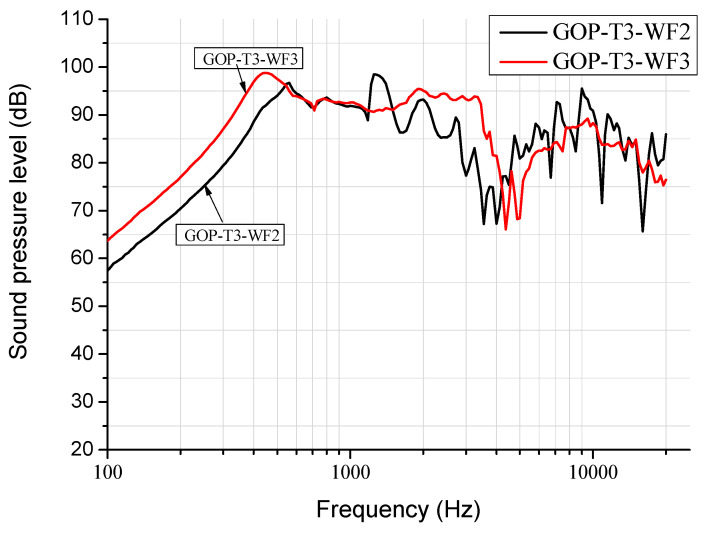
SPL curves for GO paper diaphragm headphones fabricated by various GO weight fractions in suspension.

**Figure 10:**
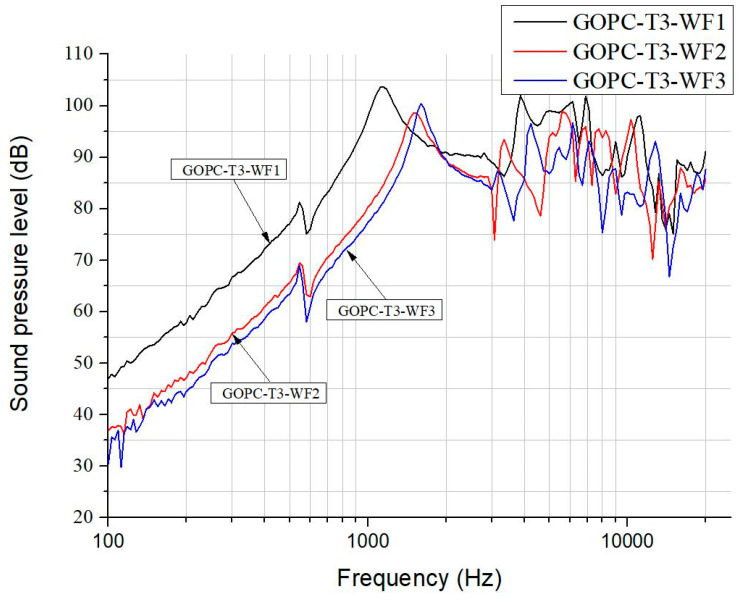
SPL curves for GOP/epoxy diaphragm headphones fabricated by various GO weight fractions in suspension.

Figure 9 illustrates the SPL curves for GOP diaphragm headphones fabricated by various GO weight fractions in suspension. According to our studies, a higher weight fraction of WF3 can lead to thicker GOP with higher compliance which results in the GOP-T3-WF3 headphone, with a larger bandwidth around 500 Hz, a smoother curve around 10 kHz, and a higher maximum SPL value of 98 dB at 500 Hz, as compared with that by WF2. In contrast, Figure 10 depicts a different trend of the effect of GO weight fraction in suspension on SPL curves for GOPC diaphragm headphones as compared with Figure 9. Among these three headphones, the lowest weight fraction of WF1 leads to GOPC-T3-WF1 with the highest SPL value of 104 dB, and the best bandwidth at 1500 Hz. This is because a lower weight fraction of WF1 can lead to thin GOP where epoxy resin is easier to infiltrate, and this further results in better interlayer bonding of GOPC.

Based on the results in reference [12], the addition of graphene in the epoxy resin would reduce its toughness. Therefore, it can be inferred that the toughness of GOPC is higher than that of GOP, and the resonant frequency of GOPC is also higher than that of GOP because the frequency is proportional to the toughness value. This is verified in Figure 9 and Figure 10, in which the first resonant frequencies of GOP-T3-WF3 and GOPC-T3-WF3 were 450 Hz and 1500 Hz, respectively. Another result [12] showed that the damping ratio of GNPs-reinforced composites was higher than neat epoxy and proportional to the weight fraction of GNP addition. According to the rule of mixtures, it is inferred that a single graphene nanosheet has a higher damping ratio than the epoxy. However, due to non-uniformity and pores inside the laminate GOP, it could be assumed that the damping ratio of GOPC is higher than that of GOP in our work. A higher damping ratio could be beneficial to reduce transient distortion; in other words, the headphone could perform a smoother SPL curve at middle to higher-frequency range because upon frequency sweeping the vibration of the diaphragm could quickly reduce to its original position. This can be proved in Figure 3 that at around 4–5 kHz the SPL curves for GOP-T3 have a significant drop; while the drop for GOPC-T3 is flatter. This drop probably can be better adjusted by using a different designing headphone unit (case) as shown in Figure 1d.

## 4. Conclusions

In this paper, four kinds of diaphragm headphones are fabricated including GOP (graphene oxide paper), GOPC (GOP/epoxy nanocomposites), GOPC-L (Line indented GOPC), and GOPC-C (Curve indented GOPC) headphones, and the influencing factors on their SPL performance are investigated. Several interesting findings are summarized as follows. Results show that the GOP diaphragm headphone possessed a wider bandwidth and a smoother SPL curve in a lower frequency range, a high initial SPL value of 71 dB at a sweeping frequency of 100 Hz, and a good SPL value of 99 dB at the first resonant frequency of 430 Hz. On the other hand, the GOPC diaphragm headphone has a comparative smoother SPL curve in a higher frequency range, a lower initial SPL value of 30 dB at 100 Hz, and the highest SPL value of 104 dB. The higher initial SPL value of GOP is caused by its high compliance value. In addition, even without the addition of epoxy, a pure GOP diaphragm can achieve a maximum SPL of 99 dB due to its high specific modulus. A higher damping ratio and lower compliance of 0.3 m/N result in the GOP diaphragm with flatter SPL curve at higher frequency. Both indented patterns on GOPC seem to improve initial SPL values at the lowest frequency of 100 Hz due to the increase in compliance. In addition, the patterns lead to smoother SPL curves for frequencies higher than 10 kHz because of the more uniform microstructure.

Owing to the different fabrication features of GOP and GOPC, the effects of sonication time and graphene weight fraction on their SPLs are in reverse. Both T1 and WF3 lead to the GOP with better SPL because they all provide better interlocked 2D nanosheets for a good diaphragm. On the other hand, both T3 and WF1 result in the GOPC with better SPL because they provide a more uniform microstructure for the composite. To sum up, GOP and GOPC diaphragms could be high potential candidates for headphones applied in lower- and higher-frequency ranges, respectively. Therefore, GOP and GOPC diaphragms may be synergistically combined into a high-performance two-way headphone.

## Figures and Tables

**Table 1:** Terminology for various graphene diaphragms.

Diaphragm Type	Sonication Time on Suspension (min)	GO Weight Fraction in Suspension (wt%)	Pattern Type on Nanocomposite
GOP	T1 (1)	WF1 (0.5)	Lined (GOPC-L)
(Graphene oxide paper)	T2 (5)	WF2 (1.0)	Curved (GOPC-C)
GOPC	T3 (10)	WF3 (1.5)	
(GOP/epoxy nanocomposite)			

**Table 2:** Compliance of various diaphragm headphones.

T3-WF3	Compliance C_ms_ of Various Diaphragm Headphones			
	GOP	GOPC	GOPC-L	GOPC-C
Cms (m/N)	7.25	0.30	1.14	0.88

**Table 3:** Maximum SPL values of various diaphragm headphones.

Maximum SPL (dB)			
GOP	GOPC	GOPC-L	GOPC-C
99	104	101	103

## Data Availability

Data are contained within the article.

## References

[1] Dikin D.A., Stankovich S., Zimney E.J., Piner R.D., Dommett G.H.B., Evmenenko G., Nguyen S.T., Ruoff R.S. (2007). Preparation and characterization of graphene oxide paper. Nature.

[2] Suk J.W., Kirk K., Hao Y., Hall N.A., Ruoff R.S. (2012). Thermoacoustic sound generation from monolayer graphene for transparent and flexible sound sources. Adv. Mater..

[3] Xu S.C., Man B.Y., Jiang S.Z., Chen C.S., Yang C., Liu M., Gao X.G., Sun Z.C., Zhang C. (2013). Flexible and transparent graphene-based loudspeakers. Appl. Phys. Lett..

[4] Brzhezinskaya M., Irzhak A., Irzhak D., Kang T.W., Kononenko O., Matveev V., Panin G., Roshchupkin D. (2016). Direct growth of graphene film on piezoelectric La_3_Ga_5.5_Ta_0.5_O_14_ crystal. Phys. Status Solidi—Rapid Res. Lett..

[5] Kim C.S., Lee K.E., Lee J.M., Kim S.O., Cho B.J., Choi J.W. (2016). Application of n-doped three-dimensional reduced graphene oxide aerogel to thin film loudspeaker. ACS Appl. Mater. Interfaces.

[6] Ren S., Rong P., Yu Q. (2018). Preparations, properties and applications of graphene in functional devices: A concise review. Ceram. Int..

[7] Mehrez S., Karati S.A., DolatAbadi P.T., Shah S.N.R., Azam S., Khorami M., Assilzadeh H. (2020). Nonlocal dynamic modeling of mass sensors consisting of graphene sheets based on strain gradient theory. Adv. Nano. Res..

[8] Jiang S., Shi S., Wang X. (2014). Nanomechanics and vibration analysis of graphene sheets via a 2D plate model. J. Phys. D Appl. Phys..

[9] Garcia-Sanchez D., van der Zande A.M., Paulo A.S., Lassagne B., McEuen P.L., Bachtold A. (2008). Imaging mechanical vibrations in suspended graphene sheets. Nano. Lett..

[10] Sakhaee-Pour A., Ahmadian M.T., Naghdabadi R. (2008). Vibrational analysis of single-layered graphene sheets. Nanotechnology.

[11] Mirakhory M., Khatibi M.M., Sadeghzadeh S. (2018). Vibration analysis of defected and pristine triangular single-layer graphene nanosheets. Curr. Appl. Phys..

[12] Rafiee M., Nitzsche F., Labrosse M.R. (2019). Processing, manufacturing, and characterization of vibration damping in epoxy composites modified with graphene nanoplatelets. Polym. Compos..

[13] Jamal-Omidi M., ShayanMehr M. (2018). An experimental study on the nonlinear free vibration response of epoxy and carbon fiber-reinforced composite containing single-walled carbon nanotubes. J. Vib. Control.

[14] Rafiee M., Nitzsche F., Labrosse M.R. (2018). Modeling and mechanical analysis of multiscale fiber-reinforced graphene composites: Nonlinear bending, thermal post-buckling and large amplitude vibration. Int. J. Non-Linear Mech..

[15] (2003). Sound System Equipment—Part 5: Loudspeakers.

[16] Liu H.K., Hwang S.F., Yang S.H., Lei C.H. (2020). Fatigue analysis of graphene oxide papers fabricated under various processing parameters. Fatigue Fract. Eng. Mater. Struct..

[17] Zhang L., Alvarez N.T., Zhang M., Haase M., Malik R., Mast D., Shanov V. (2015). Preparation and characterization of graphene paper for electromagnetic interference shielding. Carbon.

[18] Kang D.J., Chen F., Park J.H. (2014). New measurement method of Poisson’s ratio of thin films by applying digital image correlation technique. Int. J. Precis. Eng. Manuf..

